# Three‐dimensional stem cell models of mammalian gastrulation

**DOI:** 10.1002/bies.202400123

**Published:** 2024-08-28

**Authors:** David A. Turner, Alfonso Martinez Arias

**Affiliations:** ^1^ Institute of Life Course and Medical Sciences, William Henry Duncan Building, Faculty of Health and Life Sciences University of Liverpool Liverpool UK; ^2^ Systems Bioengineering, DCEXS Universidad Pompeu Fabra Barcelona Spain

**Keywords:** 3D models, embryonic stem cells, gastrulation, gastruloids, organoids

## Abstract

Gastrulation is a key milestone in the development of an organism. It is a period of cell proliferation and coordinated cellular rearrangement, that creates an outline of the body plan. Our current understanding of mammalian gastrulation has been improved by embryo culture, but there are still many open questions that are difficult to address because of the intrauterine development of the embryos and the low number of specimens. In the case of humans, there are additional difficulties associated with technical and ethical challenges. Over the last few years, pluripotent stem cell models are being developed that have the potential to become useful tools to understand the mammalian gastrulation. Here we review these models with a special emphasis on gastruloids and provide a survey of the methods to produce them robustly, their uses, relationship to embryos, and their prospects as well as their limitations.

## Graphical abstract

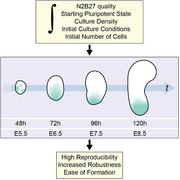

## INTRODUCTION

In all organisms, fertilization of an egg triggers a process of cell proliferation leading to an ensemble of seemingly identical cells that progressively segregate into distinct lineages. In amniote vertebrates (reptiles, birds, and mammals) one of the first lineages gives rise to the epiblast, often a circular epithelium that will become the embryo proper. As development progresses, a conspicuous furrow can be observed along the midline of the epiblast that presages the anteroposterior (AP) axis of the organism. The furrow, called the primitive streak, signals the start of the process of gastrulation and is associated with an epithelial mesenchymal transition (EMT) that acts as a conduit for a process of directional cell migration. Gastrulation transforms the epiblast disc into a complex three‐dimensional structure within which the three germ layers (ectoderm, mesoderm, endoderm) organized with reference to a coordinate system that also acts as a reference for the arrangement of the primordia of tissues and organs. At the end of gastrulation, the initial mass of cells that results from fertilization has been sculpted into the outline of an organism, its body plan.^[^ ^1^ ^]^

Gastrulation is conditioned by variations in the structure of the egg across different species. Reptiles and birds are oviparous, develop within yolk‐rich eggs that are laid externally and hatch at the end of development. In these animals, fertilization leads to a very large epiblast with thousands of cells and gastrulation is, principally, a matter of wholesale cell orientated movements. On the other hand, mammals are viviparous, develop inside the mother from eggs with very little yolk, and their embryos rely on maternal supplies of nutrient for their growth and patterning. In this case, gastrulation takes place after two lineage decisions that specify extraembryonic tissues that will link the embryo to the mother, the placenta, or contribute to its early nourishment, the extraembryonic endoderm. At the onset of gastrulation mammalian embryos are made up of no more than about 300 cells and this contrasts with the thousands of frogs, fish, and chickens. This means that mammalian gastrulation takes place against a background of vigorous cell multiplication^[^ ^2^ ^]^ that is running in parallel with a dramatic process of self‐organization.

Advances in microscopy and embryo culture have opened up the possibility of studying gastrulation in the mouse^[^ ^3^ ^]^ and further developments in the long‐term culture of mouse embryos *ex vivo* have opened up possibilities for experimentation.^[^ ^4^ ^]^ However, the small litter sizes and the delicate nature of the embryos still create challenges for experimental studies, particularly in the period leading up to gastrulation. In the case of primate embryos, it has been possible to culture them through this time until the end of gastrulation, but the current protocols produce embryos that are not developing normally.^[^ ^5^, ^6^ ^]^ In the case of human embryos, it has also been possible to grow them until gastrulation (Day 14),^[^ ^7^, ^8^, ^9^ ^]^ a stage that represents a legal limit for laboratory work with human embryos in many jurisdictions, though again, under the current conditions the embryos do not look normal.

Over the last few years pluripotent stem cells (PSCs) have led to a number of in vitro systems that allow an experimental study of early mammalian development.^[^ ^10^ ^]^ These systems have the potential of assisting work in early development in a manner that, at the moment, embryos cannot. However, we surmise that for a PSC model to be deemed a model of gastrulation, it has to fulfil one or more of a number of requirements: it has to break symmetry and, in three dimensions, acquire the coordinate system that acts as a reference for the organization of organ primordia or, at least a polarization with an AP axis. Additionally, it has to generate in a spatio‐temporally organized manner the fates that characterize the mammalian body plan.

Here we discuss these models in this light with a major focus on the *gastruloid* system, a PSC model of both gastrulation and the emergence of the body plan. Our aim is to look critically at methods in their generation, their biological meaning, experimental limitations, and future prospects of these embryo models.

## Cell fate choices in a dish

The primary building blocks of embryo models are PSCs. There are several kinds of PSCs that span a continuum of states between the blastocyst and gastrulation.^[^ ^11^ ^]^ Embryonic stem cells (ESCs) are derived from the blastocyst (ESCs, naïve) and epiblast (EpiSCs, primed) of mammalian embryos and are pluripotent that is, have the ability to give rise to all cell types of an organism and maintain this ability indefinitely in culture. A third kind of PSCs are induced PSCs (iPSCs), that are derived from the reprogramming of differentiated cells;^[^ ^12^, ^13^ ^]^ in mouse these cells are in a naïve state, whereas in human, they are in a primed state. Human ESCs are equivalent to mouse primed cells (EpiSCs), though they can be converted to a naïve state with chemical treatment;^[^ ^14^ ^]^ surprisingly, naïve human cells are totipotent that is, they have the capacity to give rise to the three blastocyst lineages.^[^ ^15^ ^]^

The discovery that PSCs can be differentiated in culture toward, in principle, any cell type was important to develop different models of embryonic development. In particular, detailed analysis of the paths of differentiation revealed that, upon exiting pluripotency, PSCs go through a sequence of stages that mimic gastrulation and express markers of the primitive streak before specific differentiation markers.^[^ ^16^, ^17^, ^18^ ^]^ This can be achieved in adherent culture under specific signals that are tailored to particular fates.

In a population of PSCs under self‐renewal conditions, only a small proportion of cells exist in a competent state to respond to differentiation signals and, in general, differentiation proceeds in an asynchronous and heterogeneous manner.^[^ ^18^, ^19^, ^20^ ^]^ A study of the reasons for this behavior led to the realization that, to differentiate, cells need to exit the pluripotent state and enter what appears to be a holding state,^[^ ^21^ ^]^ probably similar to the formative state of pluripotency. This observation is likely to reflect the situation in the postimplantation mouse epiblast where cells maintain pluripotency in a primed state for 2 days behaving as a proper stem cell population with self‐renewal and differentiation.^[^ ^22^ ^]^

In addition to a temporal progression of the exit of pluripotency, there might be aspects concerning the organization of the cells. To address this, Warmflash and colleagues constrained human PSCs in micropatterns.^[^ ^20^ ^]^ In these arrangements, subject to the signals that initiate gastrulation, cells seeded in tight circular patterns differentiate in a spatially organized concentric rings that are identified as elements of the three germ layers. From the inside out: ectoderm, mesoderm, endoderm, and extraembryonic cell types. This configuration has been extremely useful to understand the way PSCs respond to signals early in differentiation and, to a lesser degree, how they organize in space^[^ ^23^ ^]^ This experimental system has also allowed the exploration of mechanical cues into the fate choice system.^[^ ^24^ ^]^

## Mouse gastruloids

While it is possible to obtain many different cell types in adherent culture differentiation, albeit in a heterogeneous manner, the acquisition of some fates requires an initial period of aggregation. Initially, this was achieved through the embryoid body (EB) protocol, where a tight three‐dimensional aggregate of PSCs is formed in suspension culture from (usually) many thousands of cells.^[^ ^25^ ^]^ In many instances, EBs exhibit a localization of Brachyury/TbxT (TbxT) expression to a pole in the aggregate^[^ ^26^, ^27^ ^]^ after which the system becomes disorganized. While cells continue to differentiate, they do so in a heterochronic and spatially disorganized manner. Together with the results from micropatterns, these observations emphasized the point for need, patterned interactions between cells, as well as a control of their physical environment, for the coordination of their differentiation and, perhaps also for the interpretation of chemical signals.

**FIGURE 1:**
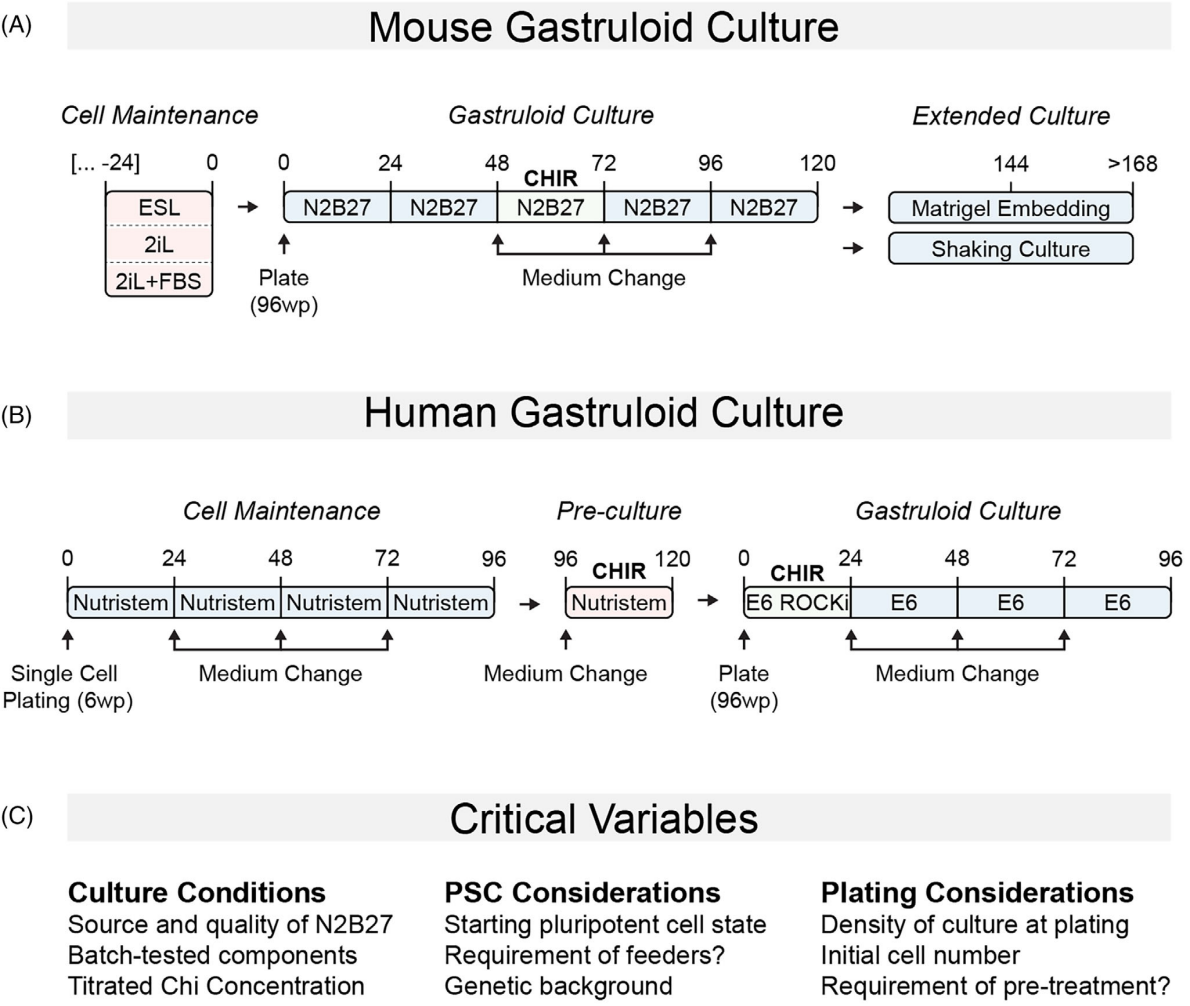
General workflow for generating mouse and human gastruloids: (A) Mouse embryonic stem cells are maintained in conditions that maintain their pluripotency (e.g., ESL: serum and LIF; 2iL; 2iL+serum). Approximately 300 cells are seeded into 96‐well plates in the basal N2B27 medium. Aggregates are exposed to a 24 h pulse of CHIR between 48 and 72 h, and maintained in culture until 120 h. If extended culture is required, they can be “shaken” or embedded in Matrigel. (B) Prior to seeding, a subculture of human ESCs are plated in 6‐well plates from a single cell suspension in Nutristem for 96 h, after which the forming colonies are exposed to a pulse of CHIR for 24 h. Cells are then dissociated to single cells and plated in E6 medium containing CHIR and ROCKi for 24 h, followed by culture in E6 medium. (C) A number of critical factors that are important for both mouse and human gastruloids are listed. Batch‐tested components are essential, as is the quality and source of N2B27. A‐P indicates the anteroposterior axis.

**FIGURE 2:**
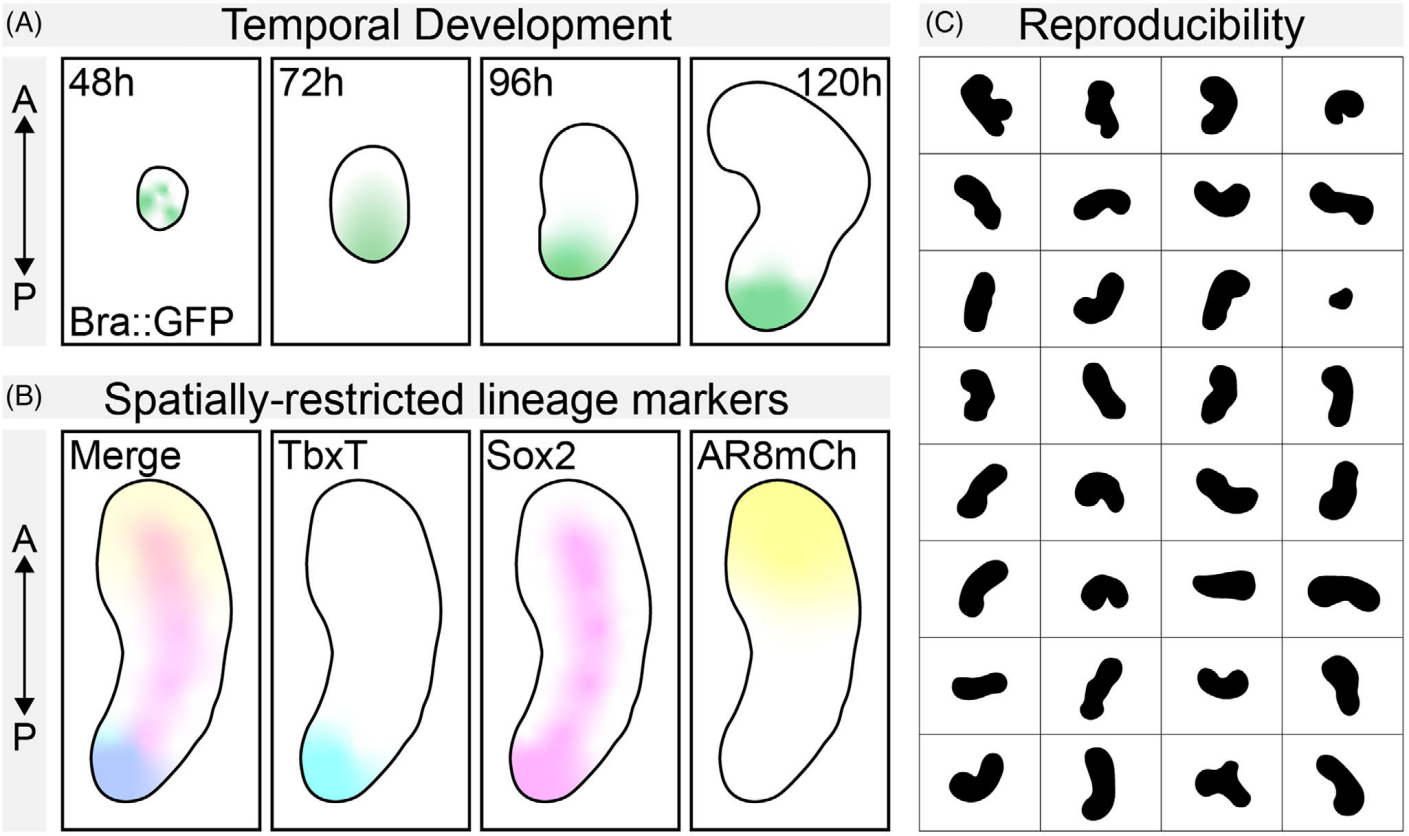
Temporal and spatial reproducibility of Gastruloids. (A) A schematic example of gastruloids formed from Bra::GFP mouse embryonic stem cells over time. Note the early symmetry‐breaking, polarization of GFP, and the axial elongation over time. (B) Stereotypical polarization and patterning of specific markers in a typical 120 h gastruloids showing Tbxt (cyan), Sox2 (magenta), and a BMP signaling (yellow). (C) Typical brightfield images of E14Tg2A mouse ESCs at ∼120 h showing a high degree of reproducibility within a single experiment. Note the smooth elongating region (which would be TbxT positive).

In 2009, Yusuke Marikawa reported that, upon release from pluripotency and in the presence of Serum, aggregates of embryonal carcinoma (EC) cells organize themselves into elongated structures that resemble amphibian exogastrulae.^[^ ^28^ ^]^ Furthermore, in these structures, the extending mass displays localized expression of many tail bud genes, including *TbxT*, suggesting that they represent the posterior epiblast of the mouse embryo. Interestingly, application of this simple protocol to ESCs did not yield similar results (Y.M. personal communication and A.M.A. unpublished observation). Extension of this observation to mouse ESCs required precise modification of the experimental conditions, drawing on our (and other's) observations from adherent culture and differentiation of PSCs.^[^ ^18^, ^29^ ^]^ The result is the *gastruloid* system (Figure 1, 2).

In the current standard protocol, defined numbers of mouse ESCs are aggregated in neurobasal medium (N2B27) and, after 48 h, exposed to high levels of the Wnt agonist CHIR99021 (Chiron). At 120 h after aggregation (AA), a structure emerges with a prominent elongated side and a dense core at the opposite end.^[^ ^21^, ^30^ ^]^ Gene expression analysis of these structures revealed a patterned arrangement of cell types that, at 120 h, resembled the E8.5 mouse embryo but in the absence of extraembryonic tissues and anterior neural cell types.^[^ ^30^, ^31^, ^32^ ^]^

## Mouse gastruloids: Principles and variables

Most gastruloid work to date has involved mouse PSCs, and current protocols can attain frequencies of more than 90% success from starting cultures, see for example, in refs. [32, 33, 34]. This makes the system amenable to detailed measurements and large‐scale screens,^[^ ^34^ ^]^ as well as to single gastruloid studies.^[^ ^35^ ^]^ However, our own experience and that from several other labs has revealed that the gastruloid protocol is highly sensitive to small changes in a number of variables that need to be precisely controlled. Due to the central importance of these conditions, we will summarize and comment on them here (Figure 1C).

*Starting pluripotent cell state*. Mouse gastruloids can be made with naïve ESCs and iPSCs. The current protocol does not work with EpiSCs or other states of pluripotency.^[^ ^36^ ^]^ The culture conditions of PSCs before aggregating in gastruloid formation is critically important. The initial protocols used Serum and LIF to culture PSCs, but many labs use 2i/LIF and even 2i/Serum/LIF to grow cells; this reduces the heterogeneity of the starting population and delays elongation (unpublished observation). A recent report has analyzed in detail the effect that the growth conditions of the PSCs have on the cell type diversity of the gastruloids.^[^ ^37^ ^]^ Growth on feeders has been shown to produce good yields and homogeneities in gastruloid formation.^[^ ^36^ ^]^ Furthermore, we and others have observed that the cell line, and probably its genetic background, can make a difference to whether a gastruloid forms or not.

*The density of the culture* at the time of aggregate formation also appears to matter. In many instances, a culture in Serum/LIF might drift to differentiation. In this instance, it is advisable to give cells one or two passages in 2i/LIF before starting the gastruloid protocol. The concentration of CHIR99021 (CHIR; Wnt/β‐Catenin agonist, GSK3 antagonist) required to trigger gastruloid formation might need to be adjusted from one cell line to another.

*The number of initial cells* appears to be particularly critical and should be tailored for each cell line. The emergence of a gastruloid with a single polar axis requires an initial number of cells between 100 and 400 cells. Too few cells and gastruloids will form in a stochastic manner, slightly too many starting cells and gastruloids with multiple axes emerge, and starting with far too many cells disorganized EBs will develop.^[^ ^30^, ^33^ ^]^ For most cell lines, the crucial number appears to be around 300. There appears to be a correspondence between the initial number of cells and the final size of the gastruloid and the patterns of gene expression scale over a range of sizes.^[^ ^33^ ^]^

*Culture conditions*. A highly important variable in gastruloid formation is the quality of the gastruloid growth medium, N2B27. There seems to be a variability in the commercial medium whose origin is difficult to pinpoint, but many labs make their own. Interestingly, the crucial ingredient here seems to be the source of the N2 supplement, and batch testing (using the ability to form elongated gastruloids as a readout) is essential.^[^ ^38^ ^]^

When conditions are optimal for reproducible and robust gastruloid formation, analysis of gene expression over time suggests that, at 48 h, most of the cells in the aggregate have exited pluripotency and are in a state where they can respond to signals.^[^ ^18^, ^29^, ^31^, ^32^, ^34^ ^]^ At this time, the aggregate expresses epiblast genes and exposure to a Wnt signaling agonist, in the form of Chiron, triggers events between 48 and 72 h AA that mimics gastrulation, as assessed by emergence of different cell types and polarization of gene expression along an AP axis.^[^ ^30^, ^31^, ^32^, ^34^ ^]^

Gastruloids lack anterior neural cell types. This is likely due to the exposure of the aggregates to high levels of Wnt pathway activation, which is known to suppress the development of anterior neuroectoderm.^[^ ^39^ ^]^ Exposure of the aggregate to inhibitors of Wnt signaling^[^ ^40^ ^]^ or the presence of extraembryonic endoderm^[^ ^41^ ^]^ leads to gastruloids with the addition of anterior neural structures. An example of the relationship between exposure to Wnt activation and anterior neural emergence can be observed in an adaptation of the standard gastruloid protocol.^[^ ^42^ ^]^ In this system, a small aggregate of PSCs is exposed to BMP, and begins to express *Wnt3* and *Nodal* and, later *TbxT*. Apposition of this aggregate to a larger one that is just leaving pluripotency, leads to a polarized expression of *TbxT* at the juxtaposed end of smaller aggregate, with the most anterior cells expressing hindbrain markers. In this system, Wnt pathway activity is polarized because only the smaller induced aggregate was exposed to BMP‐dependent Wnt activation, and the exposure to Wnt signaling decays from the fusion point, leading to the development of anterior neural fates furthest from the initiating aggregate.

In summary: (1) gastruloids are easy to form, robust and reproducible; (2) The quality of N2B27 medium is paramount; (3) CHIR is essential. In this piece, we shall refer to this protocol as the “classic protocol” where patterning is triggered by exposure to CHIR.

## Mouse gastruloids: Interpretation

Analysis of the patterns of gene expression in gastruloids at 120 h AA from different laboratories has shown a fair degree of consistency and reproducibility in cell fate complexity and spatial organization. The expression of *Hox* genes suggests an AP axis with the extending region being a faithful representation of the axially extending part of the embryo, with an overrepresentation of paraxial mesoderm and spinal cord tissues.^[^ ^31^, ^43^, ^44^, ^45^ ^]^ This suggests the presence of neuromesodermal progenitors (NMPs) at the posterior tip, that also contains a structure that resembles the node.^[^ ^21^, ^31^, ^45^, ^46^, ^47^ ^]^ In the anterior region of the gastruloid, there is a more variable, and less organized ensemble of cell types, that appear to be a mixed collection of progenitors of cardiac, neural crest, craniofacial and hemogenic mesoderm.^[^ ^44^ ^]^ Gastruloids also contain primordial germ cells (PGCs) that associate with different tissues throughout the protocol, perhaps indicative of cell migration.^[^ ^44^, ^45^, ^48^ ^]^

The presence and degree of endoderm in the classic protocol can vary from one cell line to another.^[^ ^44^ ^]^ This suggests that different cell lines might have intrinsically different levels of Nodal signaling, which is the main inducer of endoderm although some level of Nodal signaling is required for gastruloid formation and development.^[^ ^32^, ^36^ ^]^ Addition of Activin, as a surrogate for Nodal, together with CHIR increases the frequency and amount of endoderm.^[^ ^49^ ^]^ Notably, growth in hypoxia conditions creates endoderm and normal gastruloid development, even in the absence of CHIR.^[^ ^50^ ^]^

A recent series of important studies have shown that the axial organization of the mouse embryo is divided into two modules dependent on different T‐box factors. One is organized by Eomesodermin (Eomes), corresponds to anterior fates (endoderm, cardiac, craniofacial and hemogenic mesoderm) and requires Nodal signaling, while a second module (more posterior, activated later) depends on TbxT, and requires Wnt signaling.^[^ ^51^, ^52^, ^53^ ^]^ We suspect that the classic gastruloid system reflects the TbxT dependent module, with a variable contribution of the Eomes dependent network depending on the amount of intrinsic Nodal expression expressed by the cells that, in turn, depends on several of the variables mentioned above.

Overall gastruloids do not look like embryos despite having embryo‐like spatially organized and proportioned domains of gene expression. This suggests the absence of some morphogenetic events in gastruloids. One conspicuous example of a disconnect between genetic and morphogenetic activities is observed in the patterning of the paraxial mesoderm. In suspension culture, gastruloids exhibit axially organized patterns of gene expression associated with somitogenesis but no somites.^[^ ^31^ ^]^ Exposure to Matrigel elicits the emergence of somites in the region where somite gene expression is confined.^[^ ^30^, ^45^ ^]^ This observation opens up the possibility of studying the relationship between gene (GRNs) and cell (CRNs) regulatory networks^[^ ^54^, ^55^ ^]^ that underlies morphogenesis. In other instances for example, the gut tube, gastruloids seem to provide the environment for primordial endodermal cells to form a tube,^[^ ^56^, ^57^ ^]^ indicating the existence of an intrinsic morphogenetic program in this tissue.

In general, it is agreed that at 120 h AA, gastruloids resemble E8.5 embryos^[^ ^30^, ^31^, ^34^, ^44^, ^45^, ^58^ ^]^ with representation of all three germ layers arranged along an axial coordinate system, but without anterior neural cell types, that is, gastruloids have undergone the process of gastrulation. However, at no time during the protocol there is a presence of a primitive streak. Cells in the gastruloid appear to be in an intermediate state between epithelial and mesenchymal^[^ ^30^ ^]^ and therefore, we would like to surmise that the starting cell state of gastruloids represents the same state as cells in the primitive streak. In this case, a gastruloid would be a “self‐organized primitive streak.” The early response to CHIR with the whole gastruloid expressing *TbxT* ^[^ ^34^, ^47^ ^]^ and the later organization into different primordia support this contention. This suggestion would explain the absence of a primitive streak in gastruloids as they represent the primitive streak.

At the moment, gastruloids have been shown to represent a number of events associated with gastrulation and in some instances (e.g., somitogenesis), early organogenesis; in doing so, opening up new avenues for research (Table 1). A most striking feature of these studies is that they suggest a modular nature in the construction of the embryo. Gastruloids might be useful to reveal the components of each module, for example, it is possible to observe paraxial mesoderm without a significant representation of lateral or intermediate mesoderm or one can ask the question of what features are added to the differentiation of the paraxial mesoderm by the presence of a spinal cord. It will be interesting to look into this in some detail to see what we learn about the units and their relationships. The possibility of generating chimeric gastruloids^[^ ^59^ ^]^ adds one more experimental component to the exploration of this modularity.

**TABLE 1:** An overview of studies using gastruloids and the processes and/or cell types they can produce.

Process/Cell type	Species	References		Comments
Axial extension	Mouse	Beccari et al. (2008)	[31]	Genetic characterization of gastruloids in space and time.
Axial extension	Mouse	Rekaik et al. (2003)	[43]	Hox temporal expression. control by CTCF.
Axial extension	Human	Hamazaki et al. (2024)	[58]	Extended gastruloids and role of retinoic acid in axial organization.
Axial extension	Human	Yaman et al. (2023)	[109]	Engineered gastruloids reveal role of gradients in somitogenesis.
Axial extension	Human	Anand et al. (2023)	[110]	Engineered gastruloids reveal excitable systems in somitogenesis.
Biophyisics	Mouse	Gsell et al. (2023)	[111]	Tissue flows and symmetry breaking.
Blood	Mouse	Rossi et al. (2022)	[112]	Development of hemogenesis
Blood	Mouse	Ragusa et al. (2022)	[80]	Hemogenic gastruloid and modelling of infant leukemia.
Brain	Mouse	Girgin et al. (2021)	[40]	Neural biased gastruloids and he role of Wnt signaling on anterior development.
Brain	Mouse	Berenger‐Currias et al. (2022)	[41]	Combining XEN and ESC in classic protocol leads to brain development.
Cardiac	Mouse	Rossi et al. (2021)	[79]	Cardiogenesis.
Cardiac	Mouse	Agiro et al. (2023)	[113]	Cardiopharyngeal progenitors.
Cardiac	Human	Olmsted (et al. (2022)	[114]	Alternative gastruloid protocol, cardiogenesis.
Endoderm	Mouse	Vianello et al. (2021)	[56]	Emergence of endoderm in gastruloids and its relationship to the embryo.
Endoderm	Mouse	Pour et al. (2022)	[115]	Endoderm specification.
Endoderm	Mouse	Farag et al. (2023)	[57]	Emergent dynamic of endoderm specification and morphogenesis.
Endoderm	Mouse	Hashmi et al. (2022)	[49]	Endoderm specification, cell movements and morphogenesis.
Endoderm	Human	Olmsted et al. (2021)	[116]	Neuroendodermal gastruloids.
Epigenetics	Mouse	Braccioli et al. (2022)	[117]	Epigenetics in classic gastruloids.
Epigenetics	Mouse	Zeller et al. (2024)	[37]	Multiomics technology.
FGF	Mouse	Gharibi et al. (2020)	[118]	FGF signaling and pluripotency.
Gastrulation	Mouse	Xu et al. (2014)	[42]	Directed gastruloid organization. Signaling.
Gastrulation	Mouse	Mayran et al. (2023)	[119]	EMT in gastrulation. Snai, Cdh2 mutants.
Gastrulation	Mouse	Underhill et al. (2023)	[120]	ERK and FGF signaling in gastrulation and axial extension.
Gastrulation	Mouse	McNamara et al. (2023)	[121]	Signal recording and cell fates during gastrulation. Wnt and nodal.
Gastrulation	Mouse	Hennessy et al. (2023)	[122]	Retinoid acid and brachyury during gastrulation.
Gastrulation	Human	Stronati et al. (2022)	[70]	YAP in human gastruloids.
Gastrulation	Human	Moris et al. (2020)	[69]	Establishment and characterization of human gastruloids.
Gastrulation	Mouse	Wehmeyer et al. (2022)	[59]	Mosaic gastruloids. Study of Brachyury and endoderm.
Gastrulation	Human	Yamanaka et al. (2023)	[123]	Extension of gastruloid protocol to human somitogenesis.
Gastrulation	Human	Miao et al. (2023)	[115]	Somitogenesis and somite formation.
Gastrulation	Mouse	de Jong et al. (2024)	[124]	Convergent extension and axial elongation.
Gastrulation	Mouse	Cermola et al. (2021)	[36]	Gastruloids as discriminants of pluripotent state.
Gastrulation	Human	Lee et al. (2024)	[58]	BMP signaling and calcium in gastrulation.
Hypoxia	Mouse	Lopez‐Anguita et al. (2022)	[50]	Role of hypoxia in early patterning of gastruloids.
Metabolism	Mouse	Dingare et al. (2024)	[125]	Glucose metabolism and FGF signaling during gastrulation.
Metabolism	Mouse	Stapornwongkul et al. (2023)	[126]	Glucose metabolism during axial extension.
Metabolism	Mouse	Villaronga Luque et al. (2023)	[127]	Phenotypic screening in trunk‐like structures reveals role of metabolism.
methods	Mouse	Blotenburg et al. (2023)	[37]	Analysis of the influence of starting conditions on complexity and patterning.
Multiomics	Mouse	Rosen et al. (2022)	[35]	Single gastruloid, single cell analysis: Variability.
Proteomics	Mouse/Human	Stelloo et al. (2024)	[128]	Proteomics: germ layers and axial elongation.
Scaling	Mouse	Merle et al. (2024)	[33]	Size dependent gastruloid pattern. Invariance and scaling. Precision.
Somites	Human	Sanaki‐Matsumiya et al. (2022)	[129]	Modeling somitogenesis.
Somites	Mouse	Umemura et al. (2022)	[130]	Interactions between somitogenesis and circadian clocks.
Somites	Human	Budjan et al. (2022)	[131]	Model for somitogenesis.
Somitogenesis	Mouse	Veenvliet et al. (2020)	[45]	Trunk‐like structures and induction of morphogenesis.
Sumoylation	Mouse	Cossec et al. (2023)	[132]	Effect of sumoylation on gastrulation.
Symmetry breaking	Mouse	Suppinger et al. (2023)	[34]	Symmetry breaking and screening for determinants of the process.
Symmetry breaking	Mouse	Turner et al. (2017)	[32]	Symmetry breaking without extraembryonic tissues. Signaling.
Symmetry breaking	Mouse	Sagy et al. (2019)	[77]	Mechanical inputs into symmetry breaking.
Symmetry breaking	Mouse	Anlaş et al. (2021)	[133]	Signaling and symmetry breaking.
TLS	Human	Gribaudo et al. (2023)	[134]	Spinal cord and trunk‐like structures.
TLS lineage	Mouse	Bolondi et al. (2024)	[46]	Lineage analysis of fate assignments and patterning in classic gastruloids.
Toxicity	Mouse	Yuan et al. (2017)	[83]	Toxicity assays.
Toxicity	Human	Marikawa et al. (2020)	[81]	Different protocol for human gastruloids and toxicology.
Toxicity	Mouse/Human	Mantziou et al. (2021)	[84]	Pilot study of gastruloids as teratology assays.
Toxicity	Mouse	Amoroso et al. (2023)	[85]	Budesonide effects on axial elongation and mesoderm specification.
Toxicity	Mouse	Huntsman et al. (2024)	[135]	Embryotoxicity assay in reference to the ICH S5(R3) guideline chemical exposure list.

*Note*: A number of gastrulation‐associated processes (e.g., symmetry‐breaking, axial extension, somitogenesis) and cell types (e.g., endoderm, blood) that can be generated using gastruloids from either mouse or human embryonic stem cells. The key references to these studies are indicated.

Abbreviations: EMT, epithelial mesenchymal transition; ESC, embryonic stem cells.

Our suggestion that gastruloids represent a model of the primitive streak indicates a capacity for self‐organization in these cells that, perhaps, does not match the current picture of fate assignments in this structure. Text books and review articles tend the present an orderly emergence of cell fates from the primitive streak^[^ ^60^, ^61^, ^62^ ^]^ but studies of individual cells reveal wayward trajectories^[^ ^63^ ^]^ and labile cell fates^[^ ^64^, ^65^ ^]^ that would be consistent with the notion of a degree of self‐organization in the primitive streak and resulting cells in the embryo.

## Human gastruloids

The reproducibility and versatility of mouse gastruloids suggested that the classic protocol could be extended to human PSCs. This would make a compelling case for gastruloids as a system to study human gastrulation, a process that currently is out‐of‐bounds for ethical reasons associated with the 14‐day rule which does not allow the culture of embryos beyond the initiation of gastrulation.

Transferring the classic gastruloid protocol from mouse to human PSCs was challenging, due to the fact that human PSCs reflecting a “primed” state of pluripotency which, as described above, is not compatible with mouse gastruloid formation.^[^ ^36^ ^]^ Likewise, the classic mouse gastruloid protocol does not generate gastruloids from human PSCs (AMA unpub). To bypass these problems, an alternative protocol was devised to form gastruloids from mouse EpiSCs that served as the basis for human gastruloids (AMA and Tina Balayo, unpub).

The protocol developed for human PSCs^[^ ^66^ ^]^ is, at the moment, more sensitive to starting conditions than that of the mouse (Figure 1B,C). Variables that are important in mouse gastruloids (e.g., media, cell state and number, CHIR concentration) become absolutely essential for optimization in human gastruloids. This is likely to be due to the importance of PSC culture prior to gastruloid formation. Also, human PSCs are a more heterogeneous population than the mouse PSCs^[^ ^67^, ^68^ ^]^ and it is likely that different culture media shift the balance of different subpopulations influencing the proportion of cells competent to form gastruloids. It would be helpful to identify which population of cells within the human PSC culture is more amenable to gastruloid formation. Notwithstanding these issues, the published protocol works well^[^ ^58^, ^69^, ^70^ ^]^ though it is important that we acknowledge that it could be made more robust.

Like all human PSC differentiation protocols, the formation of human gastruloids has an essential priming state, see for example, the protocols in refs. [71, 72, 73] without which proper differentiation does not occur. After aggregation, human gastruloids undergo morphological changes, similar to mouse gastruloids, and, by 96 h, display an elongated morphology with a spatially structured representation of primordia from the different tissues and organs and an AP polarity.^[^ ^58^, ^74^ ^]^ The extending region contains NMPs at the posterior tip and this has been used to develop a number of models of human somitogenesis (Table 1).

One important feature of human gastruloids is their relationship to human embryos. In the original report, the onset of the program of somitogenesis was used to suggest that, at 72 h, human gastruloids correspond to human embryos around 19 days postcoitum (dpc). However, this does not mean that human gastruloids develop faster than embryos. What needs to be understood when comparing PSC states between mouse and human is that, as stated above, human PSCs are in a heterogeneous state between naïve and primed, which spans over 1‐week, and contains a wide range of states with independent representations dependent on the culture media.^[^ ^68^ ^]^ The subpopulation that usually participates in gastruloid formation corresponds to one that is about 14 to 15 dpc of development that is, they are at the gates of gastrulation. This population can be seen in a plethora of studies where addition of Wnt agonists move PSCs into fate decisions associated with gastrulation,^[^ ^71^, ^72^ ^]^ and it is this population that is favored in the culture conditions we use to generate gastruloids. Given this consideration and the 24 h pretreatment stage which triggers patterns of gene expression associated with gastrulation,^[^ ^58^ ^]^ 96 h‐old gastruloids would mean 5 days from the start of the protocol. If PSCs were, as we surmise in a stage about D14/15, 96 h would mean D19/22 as suggested.^[^ ^69^ ^]^ This has also recently been corroborated in an independent study that has also made comparison with other species.^[^ ^58^ ^]^

## Gastruloids: Uses and applications

Gastruloids can be used as an experimental system to study the self‐organizing properties of PSCs within a natural context. However, their main value lies in understanding the relationship between gastruloids and embryos; what can they teach us that embryos do not or cannot? They do this through experiments that are not possible with embryos that mirror the work of experimental embryology that thrived in the first half of the 20th century.^[^ ^75^ ^]^ This work, largely performed with frogs and salamanders, involved manipulations of different regions of developing embryos to test their autonomy and interactions and led to the discovery of the organizer and neural induction. Unfortunately, the lack of genetic techniques at the time limited the understanding of these findings and the progress of the field. Until now, mammalian systems have lacked such a versatile experimental system and developmental biology has relied on genetics: gastruloids, however, allow a blending of the two.^[^ ^76^ ^]^

A number of studies already highlight their value to study fundamental questions such as spontaneous symmetry‐breaking^[^ ^47^, ^77^ ^]^ and its relationship to genetically supervised self‐assembly,^[^ ^78^ ^]^ pattern scaling in early development^[^ ^33^ ^]^ or the relationship between gene‐regulatory networks (GRNs) and CRNs in morphogenesis.^[^ ^44^, ^45^ ^]^ Most importantly, gastruloids also reveal a modularity of embryonic development which is intrinsic to all organoids. It is obvious, but perhaps worth stating, how surprising it is that primordia of different organs and tissues can be created and differentiated in isolation from other organs and tissues, and importantly in a context that is removed from *the whole embryo*. Regarding gastruloids, this is also the case with some differences, for instance, by 72 h in gastruloids, the primordia of most organs and tissues are in place with an organization relative to each other that mimics the embryo.^[^ ^44^ ^]^ This means that interactions are possible that shape what happens afterward. The current protocol veers toward the trunk, which it does faithfully in terms of the paraxial mesoderm and neural tube, whereas changes in the protocol between 48 and 72 h AA, can alter the cell fates and their relative ratios toward specific organs and tissues.^[^ ^79^, ^80^ ^]^ This can be used in the generation of primordia for different tissues and organs as a basis for organoids, as for example with cardiac or hemogenic gastruloids.

Gastruloids are not a substitute for embryos but rather a complementary experimental system that allows many experiments, and in particular screens and experimental procedures that are not possible with embryos. It is increasingly clear that they do reflect many features of embryos but in many instances, whatever they suggest should be tested, or at least related to, embryos.

A potentially important use of gastruloids is as substrates for drug screening platforms in teratology and toxicology.^[^ ^81^, ^82^, ^83^, ^84^, ^85^ ^]^ While they still need to undergo much testing before they become a favored element in this area of research, there is little question that they are better than many current animal and cellular models that are distant from human embryos.^[^ ^86^ ^]^

## Alternative embryo models of gastrulation

Gastruloids are minimal models of mammalian gastrulation that have started to reveal much about how cells build embryos. A recent report has provided some insights into the trigger of EMT in a 3D model mouse PSCs.^[^ ^87^ ^]^ However, while this system lacks most of the features of gastrulation (see above), most notably symmetry‐breaking and the sequence of fates associated with the body plan, we therefore see this more as a model of EMT than of gastrulation.

Unlike gastruloids that are made exclusively of PSCs, embryos develop surrounded by, and interacting with, extraembryonic tissues that are likely to play a role in its morphogenesis.^[^ ^88^, ^89^ ^]^ In particular, the localization of the primitive streak and the morphogenesis of the epiblast might require inputs from the trophectoderm (TE) and the primitive endoderm (PrEnd). To explore this, over the last few years, models have been built that attempt to assemble the embryonic and extraembryonic tissues in one structure. In these studies, trophectoderm stem cells (TSC) and primitive endoderm (XEN) are brought together with PSCs and allowed to interact with each other at random.

In mouse models, combinations of TSCs with PSCs form an epiblast that, in the presence of Matrigel, initiates gastrulation from a localized position at the interface TE and the epiblast, as it does on the embryo.^[^ ^90^ ^]^ Addition of XEN or PrEnd cells to this combination forfeits the requirement for Matrigel and leads to more complete structures in which a primitive streak‐like structure progresses and, sometimes, reaches the distal part of the epiblast.^[^ ^91^, ^92^ ^]^ A conclusion from these studies is that an important role of the extraembryonic endoderm is to provide a basement membrane that maintains the epithelial nature of the PSCs in the epiblast and therefore provides a substrate for EMT and the spatially organized progression of the Primitive streak. The reproducibility of these experiments is low: usually less than 10% of the starting cultures generate the desired structures. This makes it difficult to use this system to extract information about cell interactions. Nonetheless, reporting increases in frequency could be used to find elements that underlie the formation of the epiblast, as in the case of Cadherins.^[^ ^93^ ^]^

A different engineering‐inspired approach treated TSC and PSC separately to first form epithelial cysts and then bring them together to study their interactions. Under these conditions, the yield of structures is higher, and it is possible to perform experiments with proper statistical rigor inherent to larger experimental numbers.^[^ ^94^ ^]^ These combinations have shown that the size of the epiblast is critical for the containment of the primitive streak and also that the role of signaling from the TE is to bias the intrinsic symmetry breaking ability of the epiblast that is revealed in the gastruloids, independently of the PrEnd. Similarly, engineering XEN, TSC, and PSC yields insights.^[^ ^95^ ^]^

In one remarkable instance, a combination of mouse PSC with genetically induced TE and PrEnd leads, under defined culture conditions, to structures that mimic an E8.5 embryo.^[^ ^96^, ^97^ ^]^ Although these structures occur at very low frequencies, on the order of 1% from starting cultures, they are remarkable and reveal the robustness of the mouse gastrulation. Nevertheless, even the best of the structures, although superficially similar to embryos, when looked at in detail can be seen to lack the structural organization of the organs in the embryo. For example, although they exhibit beating cardiomyocyte within a cardiac primordium, as well as neural plate, both structures display differences with their embryonic correlates. Notwithstanding these differences, an important feature of these structures is the presence of a forebrain that is never found in gastruloids, nor even in some of the earlier TSC, XEN, PSC combinations. This suggests that these models will be useful to understand the requirements for the emergence of the most anterior region of the embryo and how this is coordinated with the rest of the embryo. However, for these structures to be useful, a concerted effort will be required to improve the frequency of their generation.

The emergence of a body plan with mouse PSCs has encouraged extending these experiments to human embryos. Although very similar to mouse embryos at the blastocyst stage, human embryos diverge remarkably from the moment of implantation. The peri‐implantation human embryo is a very different entity from the mouse one, and exhibits a more complex organization of the TE and the PrEnd.^[^ ^98^ ^]^ Nevertheless, combinations of human PSCs with TSC and Primitive endoderm cells exhibit a high capacity of self‐organization that is manifest in a range of structures that can be used to learn about the assembly of the postimplantation human embryo and the interactions between embryonic and extraembryonic lineages (reviewed in refs. [99] and [100]).

At the moment, studying gastrulation, the progression of the primitive streak, and the emergence of the body plant, in humans remains a challenge. Surprisingly, on their own, human PSCs can form a mature epiblast, with an amnion, polarized TbxT expression and PGCs.^[^ ^101^, ^102^, ^103^ ^]^ These structures collapse without progressing to generate a body plan. One possibility for the failure of the emerging Primitive streak to progress might be a need to be constrained by its interactions with the TE and the primitive endoderm. This needs to be explored but, in the most accurate model to date, in which the emerging structures mimicked closely the 14‐day embryo,^[^ ^104^ ^]^ gastrulation does not ensue.

One study reported progress through gastrulation, and even the emergence of the body plan, from a system composed of epiblast and primitive endoderm.^[^ ^105^ ^]^ However, the frequency of these structures is extremely low and their similarity to embryos, particularly pass Day 14, arguable. A feature of these structures is the presence of anterior neural tissue.^[^ ^105^ ^]^ This is interesting since, as in the mouse, this structure only appears in “complete” models, suggesting an interaction between the extraembryonic and embryonic tissues in the development of the forebrain at the anterior end of the embryo.

An alternative route toward a model of human gastrulation relies on extended development of blastoids in vitro. In this regard, there is one claim of gastrula stage structures from blastoids.^[^ ^106^ ^]^ However, once again, a thorough comparison with embryos, raises questions about the claim.

One of the anchors of many of the different models of gastrulation comes from single‐cell RNAseq analysis. These studies always feature a representation of the cell types present at gastrulation within an embryo. However, these studies overlook that adherent culture differentiation protocols can, and will, yield similar cell ensembles. The value of the embryo models is not the array of cell types but their arrangement and relative proportions and organizations. We support the proposal that, for gastrulation and postgastrulation models, single‐cell RNAseq should be ancillary rather than primary information. The aim should be to compare the spatial organization of the different cell types.

## Summary and future prospects

There is little doubt that stem cell models of early mammalian development represent an important new research tool that is opening up avenues for experimental study of the interactions between cells and genes in the construction of organisms. They are revealing surprising features and unexpected properties of biological systems: spontaneous symmetry breaking leading to a body plan, disconnects between morphogenesis and patterned gene expression, an unexpected degree of modularity that becomes integrated in the organism. In the case of human development, they promise to be an important tool to understand events that cannot be otherwise studied. Their versatility not only permits us to learn about embryos, but also provides a good system to study the principles underlying symmetry‐breaking, its consequences in a biological system, and its relationship to genetics.

Although this field is still in its relative infancy, and we are still learning from the work, there are interesting applications ahead. For example, it has been shown that combinations of different germ layers^[^ ^107^ ^]^ or primordia of different axial levels,^[^ ^108^ ^]^ leads to better organoids. It is therefore possible that embryo models become a ground basis for improved organoids. At the moment, gastruloids have started to show this potential but, for more complete models, there is a need to increase the frequency and the reproducibility of the system.

## AUTHOR CONTRIBUTIONS

*Initial draft*: Alfonso Martinez Arias. *Subsequent writing and editing*: Alfonso Martinez Arias and David A. Turner. *Funding acquisition*: Alfonso Martinez Arias and David A. Turner.

## CONFLICT OF INTEREST STATEMENT

David A. Turner declares no conflicts of interest. Alfonso Martinez Arias is an inventor in two patents on Human Polarised Three‐dimensional Cellular Aggregates PCT/GB2019/052670 and Polarised Three‐dimensional Cellular Aggregates PCT/GB2019/052668.
